# Association between bullying victimization and obsessive-compulsive disorder: a population-based, genetically informative study

**DOI:** 10.1038/s41380-024-02849-2

**Published:** 2024-11-23

**Authors:** Josep Pol-Fuster, Lorena Fernández de la Cruz, Kayoko Isomura, Anna Sidorchuk, Ralf Kuja-Halkola, Paul Lichtenstein, Brian M. D’Onofrio, Isabell Brikell, Henrik Larsson, Elles de Schipper, Jan C. Beucke, David Mataix-Cols

**Affiliations:** 1 Department of Clinical Neuroscience, Centre for Psychiatry Research, Karolinska Institutet & Stockholm Health Care Services, Region Stockholm, Stockholm, Sweden; 2 Department of Medical Epidemiology and Biostatistics, Karolinska Institutet, Stockholm, Sweden; 3 Department of Psychological and Brain Sciences, Indiana University, Bloomington, IN USA; 4 Department of Global Public Health and Primary Care, University of Bergen, Bergen, Norway; 5 Department of Biomedicine, Aarhus University, Aarhus, Denmark; 6 School of Medical Sciences, Örebro University, Örebro, Sweden; 7 Institute for Systems Medicine, Department of Human Medicine, MSH Medical School Hamburg, Hamburg, Germany; 8 Department of Clinical Sciences, Lund University, Lund, Sweden

**Keywords:** Psychiatric disorders, Diseases

## Abstract

The extent to which bullying victimization is associated with an increased risk of obsessive-compulsive disorder (OCD) has received little empirical attention. This longitudinal, population-based, genetically informative study examined whether self-reported bullying victimization at age 15 was associated with a clinical diagnosis of OCD in the Swedish National Patient Register and with self-reported obsessive-compulsive symptoms (OCS) at ages 18 and 24 in 16,030 twins from the Child and Adolescent Twin Study in Sweden. Using a discordant twin design, including monozygotic (MZ) and dizygotic (DZ) twins, each twin was compared with their co-twin, allowing a strict control of genetic and environmental confounding. At the population level, adjusting for birth year and sex, each standard deviation (SD) increase in bullying victimization was associated with a 32% increase in the odds of an OCD diagnosis (OR, 1.32; 95% CI, 1.21–1.44), of 0.13 SD in OCS at age 18 (β, 0.13; 95% CI, 0.11–0.16), and of 0.11 SD in OCS at age 24 (β, 0.11; 95% CI, 0.07–0.16). While associations tended to persist in the within DZ-twin comparison models, the estimates attenuated and were no longer statistically significant in the within MZ-twin comparisons. These results suggest that the association between bullying victimization and OCD/OCS is likely due to genetic confounding and therefore incompatible with a strong causal effect. Other mechanisms, such as evocative gene-environment correlations, are more plausible explanations for the observed associations.

## Introduction

Despite extensive research on the etiology of obsessive-compulsive disorder (OCD), the specific causes of this condition remain largely unknown [1, 2]. OCD is moderately heritable, with heritability estimates around 50% [3–5], suggesting a substantial contribution of environmental factors. The identification of environmental risk factors that might be amenable to prevention or early intervention strategies should be regarded as a research priority [2]. Currently, few environmental risk factors have shown to be confidently associated with the development of OCD, with the possible exception of perinatal complications [6]. The identification of such risk factors is challenging as it requires large longitudinal population-based studies and genetically informative designs to rule out familial confounding [1]. In particular, the discordant twin design, involving both monozygotic (MZ) and dizygotic (DZ) twins, is a powerful tool for inferring causality, as it provides varying degrees of control over shared genetic and environmental influences [7].

One risk factor that has received considerable attention in the mental health literature is bullying victimization. Prospective population-based observational studies suggest that experiencing bullying victimization during childhood and adolescence is associated with a subsequent risk of a range of psychiatric disorders in adulthood, particularly internalizing disorders [8–12]. A smaller number of studies have used genetically informative designs, suggesting that the association between bullying and internalizing symptoms, social anxiety, and even suicidal behavior may be causal in nature, at least in children and young adults [13–15]. Whether this potential causal effect persists into adulthood remains an open question, and the possibility that the relation between bullying victimization and later psychiatric problems may be the result of genetic confounding should not be disregarded [16].

The extent to which bullying victimization is associated with an increased risk of OCD has seldom been studied. A handful of clinical-based studies have found that individuals with OCD tend to report more bullying victimization than controls [17–21]. However, the retrospective nature of these studies and their limited sample size preclude firm conclusions. Furthermore, none of these studies employed genetically informative designs, which would help address the question of whether bullying victimization may be on the causal pathway to OCD.

In this study, we linked various Swedish registers to fill this important gap in the literature. First, we examined to what extent self-reported bullying victimization at age 15 was associated with a clinical diagnosis of OCD in the National Patient Register (NPR) and with self-reported obsessive-compulsive symptoms (OCS) at ages 18 and 24 in a large sample of twins. Second, we conducted a series of within-twin pair comparisons to assess whether the observed associations were compatible with a causal effect.

## Methods

The study was approved by the Swedish Ethical Review Authority (reference number 2020-06540). Because the study was register-based and individuals were not identifiable at any time, the requirement for informed consent was waived.

### Data sources

We used the unique personal identification number assigned to every person registered as living in Sweden [22] to link several national administrative and health registers. The Child and Adolescent Twin Study in Sweden (CATSS) is an ongoing study focused on mental and somatic health, targeting all twins born in Sweden from July 1, 1992 and onwards [23]. The first data were collected in connection with the participating twins’ 9th or 12th birthday (CATSS-9/12), with several subsequent follow-ups when the twins turn 15 (CATSS-15), 18 (CATSS-18), and 24 (CATSS-24) years. CATSS-15 and CATSS-18 consist of both parent- and twin self-reports, while CATSS-24 only includes twin self-reports. Zygosity information in CATSS is determined through DNA analyses, questionnaire data or being of opposite sex, resulting in an estimated accuracy of >98% [24]. The NPR [25] includes information on diagnoses, based on the Swedish versions of the International Classification of Diseases, eighth (ICD-8; 1969–1986), ninth (ICD-9; 1987–1996), and tenth (ICD-10; 1997–onwards) revisions, registered in inpatient hospital admissions (since 1973) and outpatient specialist care (since 2001). The Total Population Register [26] was used to extract migration data; information on emigration is available since 1961 and information on immigration since 1969. The Cause of Death Register [27] contains information on dates and causes of all deaths since 1961. Finally, the Prescribed Drug Register, which contains information on all prescribed medications dispensed across pharmacies in Sweden since July 2005 [28], was used to further identify individuals with attention-deficit/hyperactivity disorder (ADHD), following previous register-based studies [29].

### Study population

Our initial cohort consisted of 16,496 twins identified through CATSS who were born from January 1, 1993 to December 31, 2006 and completed a bullying victimization questionnaire at 15 years of age. Twins who emigrated (n = 450) or died (n = 16) between their 6th birthday and the end of the study on December 31, 2020 were excluded, resulting in a final cohort of 16,030 individuals included in the analysis (9167 twin pairs, of which 6863 were complete and 2304 incomplete pairs). From this cohort, we constructed two additional sub-cohorts based on available information on self-reported OCS at ages 18 (n = 8260) and 24 (n = 3405).

### Exposure

Bullying victimization was measured at age 15 with the Revised Olweus Bully/Victim Questionnaire (OBVQ-R), a 42-item self-reported instrument which assesses different forms of bullying victimization, including verbal, exclusion, physical, spreading false rumors, personal items stolen/damaged, threats/coercion, and harassment related to race in the past two months [30]. The OBQV is moderately correlated with peer reports of victimization and has shown to be valid and reliable across countries [31]. CATSS includes a selection of 15 items of the OBVQ-R related to bullying victimization, which showed good internal consistency in the current cohort (Cronbach α = 0.72). Ten of these items cover different forms of victimization, 3 items measure the duration and intensity of the bullying, and 2 items ask the respondents how much they like school and the number of good school friends. It has been shown that a latent factor of bullying victimization can be extracted from the first 13 items, reliably capturing victimization instances, duration, and intensity [13]. Thus, we fitted a one-factor model using factor analysis and the *factanal* function from the R package Psych. We calculated a score based on this model for each individual and standardized it to mean 0 and standard deviation (SD) 1. For descriptive purposes, we also used a binary exposure variable for bullying victimization, dichotomized as in Solberg & Olweus [32].

### Outcomes

#### Obsessive-compulsive disorder diagnosis

From the NPR, we extracted the first instance of a recorded OCD diagnosis (ICD-10 code F42) made after the age of 6, to limit the risk of diagnostic misclassification [33, 34]. The ICD-10 code for OCD in the NPR has been validated by comparing registered diagnosis and information in clinical records, showing excellent inter-rater reliability and validity [35].

#### Obsessive-compulsive symptoms

At age 18, participants completed the self-reported Brief Obsessive Compulsive Scale (BOCS) [36], which is based on the clinician-administered Yale-Brown Obsessive-Compulsive Scale [37]. Each of the 15 items is rated as never, past symptoms or current symptoms. As in previous studies [38], we excluded 3 items (hoarding, body dysmorphia, and self-harm) that do not represent the core OCD phenotype and combined the past and current categories (coded as 1), while never was coded as 0. The resulting 12-item scale (range score 0-12) showed good internal consistency in the current cohort (Cronbach α = 0.76).

At age 24, participants completed the Obsessive-Compulsive Inventory-Revised (OCI-R) [39], an 18-item scale of OCS severity in the preceding month. Items are rated on a five-point Likert scale (0 = not at all, 4 = very much). The OCI-R has good internal consistency, convergent validity, and test-retest reliability [39]. Two of the 6 original sub-scales were not administered because they capture other phenotypes (hoarding; 3 items) or because of poor psychometric properties (neutralizing; 3 items) [40]. Therefore, the resulting scale had 12 items (range score 0-48) and showed good internal consistency in the current cohort (Cronbach α = 0.85). Both BOCS and OCI-R scores were standardized to mean 0 and SD 1 for the analyses.

### Statistical analyses

The discordant twin design typically involves two steps. Initially, the exposure-outcome association is examined at the population level. If an association is observed, then it is further explored comparing twins with their co-twins. A causal effect is suggested when the association persists in both DZ and MZ twins with a similar magnitude [7]. In this scenario, genetics and shared environment are less likely to explain the observed association, making causal inference a more plausible explanation.

To examine the associations between bullying victimization and OCD/OCS at the population level, we first fitted a series of logistic (binary OCD diagnoses) and linear (continuous OCS scores) regression models in the generalized estimation equations (GEE) framework, where dependencies between observations (twins) were handled using a cluster-robust sandwich estimator (*gee* function from the R package drgee) [41]. These models were adjusted for categorical birth (3-year split categories) and sex.

Because several psychiatric disorders, including OCD, may make young people more vulnerable to bullying victimisation [42], we next repeated the main analyses after excluding individuals with records of the following psychiatric diagnoses (one diagnosis at the time) recorded before age 15: OCD, neurodevelopmental disorders, anxiety-related disorders, and depressive disorders (see Supplementary Table 1 for ICD codes). In the case of OCD, this analysis also ensured that the exposure (bullying) temporally preceded the outcome (OCD).

Finally, for the co-twin comparisons, we performed conditional logistic (binary OCD diagnoses) and conditional linear (continuous OCS scores) models in the same framework, separately for DZ and MZ pairs. The model for DZ twins was adjusted for sex.

### Complementary analyses

We also performed survival analysis of OCD diagnosis after age 15 taking time to event into account. For this analysis, a sub-cohort of 15,252 twins (8,647 twin pairs, of which 6,605 were complete and 2,042 incomplete pairs) were eligible. We followed the twins from age 15 until the first diagnosis of OCD, migration, death or end of the follow-up (December 31, 2020). We fitted Cox regression models, with age as underlying time scale (*coxph* function from the R package survival) [43], adjusted for birth year and sex. Familial clustering was accounted for by using cluster-robust standard errors. For the co-twin comparisons, we performed stratified (separately for DZ and MZ pairs) Cox regression (strata option in coxph). The DZ model was adjusted for sex.

## Results

### Cohort characteristics and descriptive results

Descriptive characteristics of the study cohort are presented in Table 1. In total, 16,030 twins were included in our final cohort. Of these, 139 (0.9%) had a diagnosis of OCD registered in the NPR. Fifty-six out of these 139 individuals (40%) received their first diagnosis of OCD before age 15. The mean (SD) age at first diagnosis of OCD was 16.3 (3.6) years. There was a higher proportion of females among those diagnosed with OCD, compared to those without the disorder (62.6% vs. 54.8%; p > 0.05).

**Table 1:** Study cohort characteristics.

Characteristics	Obsessive-compulsive disorder, n (%)	No record of obsessive-compulsive disorder, n (%)
Total	139 (0.9)	15,891 (99.1)
Sex^a^		
Females	87 (62.6)	8712 (54.8)
Males	52 (37.4)	7179 (45.2)
Birth year^a^		
1993-1994	17 (12.2)	1433 (9.0)
1995-1997	36 (25.9)	3794 (23.9)
1998-2000	39 (28.1)	3623 (22.8)
2001-2003	28 (20.1)	3663 (23.0)
2004-2006	19 (13.7)	3378 (21.3)
Bullying victimization factor^b^		
Mean (SD)	0.5 (1.7)	–0.0045 (0.9)
Bullying victimization^b,c^		
Yes	15 (10.8)	565 (3.6)
No	124 (89.2)	15,326 (96.4)
OCS at age 18 (BOCS)^b,d^		
Mean (SD)	5.6 (3.0)	1.8 (2.2)
OCS at age 24 (OCI-R)^b,e^		
Mean (SD)	14.6 (9.1)	7.8 (6.8)
Comorbid psychiatric disorders before age 15^b^		
Neurodevelopmental disorders	28 (20.1)	592 (3.7)
Anxiety disorders	9 (6.5)	82 (0.5)
Depressive disorders	10 (7.2)	109 (0.7)

*BOCS* Brief Obsessive Compulsive Scale, *OCI-R* Obsessive-Compulsive Inventory-Revised, *OCS* obsessive-compulsive symptoms, *MZ* monozygotic, *DZ* dizygotic, *SD* standard deviation.

^a^*p* values not significant from either a Student t test (continuous) or a Chi-square test (categorical) comparing OCD vs. non-recorded OCD groups.

^b^*p* values significant at p < 0.001.

^c^Cut-off for bullying victimization as in Solberg & Olweus [32].

^d^For the sub-cohort of individuals with OCS in CATSS-18.

^e^For the sub-cohort of individuals with OCS in CATSS-24.

### Bullying victimization and obsessive-compulsive disorder diagnoses

Individuals with a diagnosis of OCD, compared to those without, scored significantly higher on the bullying victimization factor (M [SD] = 0.5 [1.7] vs –0.0045 [0.9]; p < 0.001). Similarly, the proportion of bullying cases (coded as present/absent) was significantly larger in those individuals with an OCD diagnosis vs those without it (10.8% vs 3.6%; p < 0.001).

At the population level, for each SD increment in bullying victimization at age 15, there was a 32% increase in the odds of being diagnosed with OCD (OR, 1.32; 95% CI, 1.21–1.44; Table 2). The magnitude of this association was similar in female and male participants (OR for female, 1.31; 95% CI, 1.17–1.46 vs. OR for male, 1.32; 95% CI, 1.15–1.52). This association remained largely unchanged when individuals with relevant psychiatric diagnoses recorded before age 15, including OCD, were excluded from the analysis (Table 3).

**Table 2:** Analyses of the association between bullying victimization and obsessive-compulsive disorder and obsessive-compulsive symptoms.

	OR (95% CI)^a^		
	Population level	Within twin comparisons	
	Minimally adjusted^b^	Within DZ^c^	Within MZ
OCD diagnosis	**1.32 (1.21–1.44)**	0.99 (0.48–2.06) 4539 pairs (76 OCD discordant)	1.07 (0.74–1.56) 2154 pairs (34 OCD discordant)

	β (95% CI)^a^		
OCS at age 18	**0.13 (0.11–0.16)**	**0.11 (0.07–0.16)** 1989 pairs	0.03 (–0.06 to 0.12) 1003 pairs
OCS at age 24	**0.11 (0.07–0.16)**	**0.12 (0.04–0.19)** 711 pairs	0.08 (–0.02 to 0.18) 430 pairs

Note: Bold figures indicate statistically significant (p < 0.05).

*CI* confidence interval, *OCD* obsessive-compulsive disorder, *OCS* obsessive-compulsive symptoms, *OR* odds ratio, *MZ* monozygotic, *DZ* dizygotic, *SD* standard deviation, *β* regression coefficient.

^a^OR/β per 1 SD increase in the bullying victimization factor score.

^b^Model adjusted for sex and year of birth. Birth year treated as categorical variable.

^c^Model adjusted for sex.

**Table 3:** Analyses of the association between bullying victimization and obsessive-compulsive disorder and obsessive-compulsive symptoms, after excluding comorbid psychiatric disorders diagnosed before age 15 (one disorder group at a time).

		OR (95% CI)^a^		
		Population level	Within twin comparisons	
		Minimally adjusted^b^	Within DZ^c^	Within MZ
OCD diagnosis	Excluding OCD	**1.21 (1.07–1.36)**	1.25 (0.45–3.47) 4506 pairs (44 OCD discordant)	0.85 (0.49–1.47) 2140 pairs (21 OCD discordant)
	Excluding neurodevelopmental disorders	**1.26 (1.12–1.41)**	1.78 (0.70–4.54) 4218 pairs (55 OCD discordant)	0.95 (0.57–1.57) 2062 pairs (29 OCD discordant)
	Excluding anxiety disorders	**1.30 (1.18–1.42)**	1.04 (0.50–2.17) 4484 pairs (71 OCD discordant)	1.07 (0.73–1.56) 2143 pairs (31 OCD discordant)
	Excluding depressive disorders	**1.31 (1.19–1.43)**	1.07 (0.50–2.32) 4478 pairs (70 OCD discordant)	0.83 (0.48–1.44) 2128 pairs (29 OCD discordant)

		β (95% CI)^a^		
OCS at age 18	Excluding OCD	**0.13 (0.10–0.16)**	**0.12 (0.07–0.16)** 1978 pairs	0.01 (–0.08 to 0.10) 995 pairs
	Excluding neurodevelopmental disorders	**0.13 (0.10–0.15)**	**0.12 (0.07–0.16)** 1909 pairs	–0.01 (–0.10 to 0.08) 976 pairs
	Excluding anxiety disorders	**0.13 (0.10–0.16)**	**0.11 (0.06–0.16)** 1974 pairs	0.03 (–0.06 to 0.12) 999 pairs
	Excluding depressive disorders	**0.13 (0.11–0.16)**	**0.11 (0.06–0.16)** 1974 pairs	0.01 (–0.08 to 0.10) 994 pairs
OCS at age 24	Excluding OCD	**0.11 (0.07–0.16)**	**0.11 (0.03–0.19)** 706 pairs	0.08 (–0.02 to 0.18) 428 pairs
	Excluding neurodevelopmental disorders	**0.10 (0.06–0.15)**	**0.12 (0.04–0.20)** 686 pairs	0.04 (–0.06 to 0.14) 419 pairs
	Excluding anxiety disorders	**0.12 (0.07–0.16)**	**0.12 (0.04–0.19)** 709 pairs	0.08 (–0.02 to 0.18) 428 pairs
	Excluding depressive disorders	**0.11 (0.07–0.16)**	**0.12 (0.04–0.20)** 703 pairs	0.09 (–0.01 to 0.18) 428 pairs

Bold figures indicate statistically significant (p < 0.05).

*CI* confidence interval, *OCD* obsessive-compulsive disorder, *OCS* obsessive-compulsive symptoms, *OR* odds ratio, *MZ* monozygotic, *DZ* dizygotic, *SD* standard deviation, *β* regression coefficient.

^a^OR/β per 1 SD increase in the bullying victimization factor score.

^b^Model adjusted for sex and year of birth. Birth year treated as categorical variable.

^c^Model adjusted for sex.

Note: Bold figures indicate statistically significant (p < 0.05).

In the within-twin comparisons, the estimates attenuated to the null and the association between bullying victimization and OCD diagnosis was no longer statistically significant when comparing DZ (OR, 0.99; 95% CI, 0.48–2.06) and MZ (OR, 1.07; 95% CI, 0.74–1.56) twins with their respective co-twins (Table 2).

### Bullying victimization and obsessive-compulsive symptoms

A 1-SD increase in bullying victimization at age 15 was associated with an increase of 0.13 SD in OCS at age 18 (β, 0.13; 95% CI, 0.11–0.16) and of 0.11 SD at age 24 (β, 0.11; 95% CI, 0.07–0.16) (Table 2). Females tended to report slightly more symptoms both at age 18 (β for females, 0.15; 95% CI, 0.11–0.18 vs. β for males, 0.11; 95% CI, 0.07–0.15) and at age 24 (β for females, 0.14; 95% CI, 0.07–0.20 vs β for males, 0.09; 95% CI, 0.04–0.14); however, the confidence intervals overlapped. These results persisted when individuals with relevant psychiatric diagnoses recorded before age 15, including OCD, were excluded from the analyses (Table 3). While these associations persisted in the within-DZ twin pair comparisons at both age 18 (β, 0.11; 95% CI, 0.07–0.16) and age 24 (β, 0.12; 95% CI, 0.04–0.19), they attenuated and were no longer statistically significant in the within-MZ twin pair comparisons (age 18; β, 0.03; 95% CI, –0.06 to 0.12; age 24; β, 0.08; 95% CI, –0.02 to 0.18) (Table 2).

### Complementary analyses

Descriptive characteristics of the cohort used in the complementary Cox regression models are presented in Supplementary Table 2. Overall, the results (Supplementary Table 3) were similar to the main results. We found that, for every additional SD in bullying victimization, the rate of OCD increased by 22% (HR, 1.22; 95% CI, 1.09–1.37). This rate did not attenuate in the within-DZ twin pair comparison, although it lost statistical significance (HR, 1.50; 95% CI, 0.93–2.40). However, the association fully attenuated in the within-MZ twin pair analysis (HR, 0.84; 95% CI, 0.48–1.47).

## Discussion

In this longitudinal, population-based twin study, we found an association between self-reported bullying victimization at age 15 and OCD diagnoses in specialist settings. The results further extended to self-reported OCS measured in the same individuals at ages 18 and 24. These results are in line with those of the few previous retrospective studies conducted in small OCD clinical samples [17–20]. The observed associations persisted even after excluding from the analyses individuals with relevant psychiatric diagnoses recorded before the age of 15, including OCD. This suggests that the results were not merely explained by either a preceding OCD diagnosis or other relevant psychiatric disorders known to make young people more vulnerable to victimization [42]. The longitudinal association between bullying and OCD was further confirmed in our survival analysis taking time to event into account, which did not include individuals with a previous OCD diagnosis.

Importantly, however, the results of the within-twin comparisons consistently showed that the magnitude of the association between bullying victimization and OCD/OCS progressively attenuated with more stringent control of genetic confounding, and it was no longer statistically significant in MZ twin comparisons. Therefore, the results are not compatible with a strong causal effect of bullying victimization. The contribution of additive genetic effects to both bullying victimization [44] and OCD [4, 5] is well known. Based on previous research in a range of internalizing disorders [16], it would be reasonable to assume that common genetic factors may at least partially influence both phenotypes. In this scenario, the observed associations may represent evocative gene-environment correlations, whereby the presence of OCD, subclinical OCS or associated behavioral, social, and academic difficulties might evoke abusive behavior from bullies [15].

Consistent with this idea, a prior study found that several polygenic risk scores (PRS) for psychiatric disorders significantly predicted the likelihood of being bullied [45]. In that study, the association between OCD PRS and bullying was not statistically significant; however, the power of the available OCD genome-wide association data at the time was very limited. Furthermore, the Schoeler et al. [45]. study could not completely rule out the potential influence of passive gene environment correlations. An ideal approach would be to use within-family designs, as previously done for other phenotypes [46, 47]. The genotype data available for DZ twins in the Swedish Twin Registry [24], together with the latest OCD PRS [48], may provide a valuable opportunity to further understand the nature of gene-environment correlations in relation to bullying and other potential risk factors.

In clinical practice, integrating knowledge about evocative gene-environment correlations concerning bullying can lead to more nuanced discussions with individuals with OCD and their families about the complex interplay between genetic and environmental factors in this disorder. It is important to approach this information tactfully, acknowledging and validating the individual’s bullying experience and emphasizing the importance of supportive environments (e.g., working with schools to eradicate bullying behaviors) and access to evidence-based treatment, irrespective of genetic predispositions.

### Strengths and limitations

To our knowledge, this was the first study to explore the association between bullying victimization and OCD and OCS in a large population-based cohort with longitudinally collected data and using a genetically informative design. The diagnoses of OCD in the NPR are highly reliable and valid [35] and the previously validated OCS scales had good internal consistency in this study. Therefore, the study contributes to address a significant gap in our understanding of the relation between bullying victimization and OCD using sound methodology.

However, the results should be interpreted in light of some limitations. Firstly, while self-reports are commonly used and they are reliable measures of bullying victimization, they may still be susceptible to self-report bias [49]. Bullying was measured at age 15, which is a reasonable time point in adolescence to capture this phenomenon, but a proportion of young people develop OCD before that age. In our study, the mean age of diagnosis was 16 years, but a substantial number of individuals received their OCD diagnoses before age 15. We adopted several approaches to mitigate this problem, such as excluding these individuals with pre-existing diagnoses from the analyses and performing complementary Cox regression analyses taking time to event into account. Additionally, we employed measures of self-reported OCS at ages 18 and 24 in a subset of the same individuals. Reassuringly, the results were highly consistent across these approaches. Finally, some of the within-twin analyses involving binary OCD diagnoses had limited statistical power, although the main results and conclusions were supported by the OCS analyses, which were better powered.

## Conclusion

This longitudinal, population-based twin study found that bullying victimization was associated with an increased risk of OCD and OCS. However, these associations were likely due to genetic confounding and therefore incompatible with a strong causal effect. Other mechanisms, such as evocative gene-environment correlations, are more plausible explanations of the observed associations.

## Supplementary information

Supplementary material

## Data Availability

Data from the Swedish registers used in this study cannot be shared due to data protection regulations. Dr. Pol-Fuster had full access to all the data in the study and takes responsibility for the integrity of the data and the accuracy of the analysis.
