# Supplementary material for: Association between bullying victimization and obsessive-compulsive disorder: a population-based, genetically informative study
Source: Mol Psychiatry. 2024 Nov 23;30(6):2457–62. doi: 10.1038/s41380-024-02849-2 (PMC12092294; doi:10.1038/s41380-024-02849-2)
Supplement: Supplementary file 1 — Supplementary material [file 41380_2024_2849_MOESM1_ESM.docx]

**SUPPLEMENTARY TABLES**

**Supplementary Table 1.**Groups of psychiatric comorbidities and their corresponding International Classification of Diseases (ICD) codes and minimal age of diagnosis.

| **Comorbidity groups** | **Included diagnoses** | **Corresponding Swedish ICD-10 diagnostic codes** | **Minimal age of diagnosis** |
| --- | --- | --- | --- |
| **Neurodevelopmental disorders** | Autism spectrum disorders, attention-deficit/ hyperactivity disorder^a^, and Tourette syndrome and chronic tic disorder^b^ | F84, F90, F95 | Diagnosed at age 1 or above for autism spectrum disorders and 3 or above for the rest |
| **Anxiety-related disorders** | Phobic, anxiety, reaction to severe stress, and adjustment disorders | F40.0, F40.1, F40.2, F41.0, F41.1, F43 | Diagnosed at age 6 and above |
| **Depressive disorders** | Major depressive disorder, persistent mood disorder, and unspecified mood disorder | F32, F33, F34 (minus F34.0), F38, F39 | Diagnosed at age 6 and above |
| ^a^ Individuals with attention-deficit/hyperactivity disorder (ADHD) were also identified by prescription of ADHD drugs, collected from the Prescription Drug Register, specifically Amphetamine (Anatomical Therapeutic Chemical [ATC] Classification System code: N06BA01), Dexamphetamine (N06BA02), Methylphenidate (N06BA04), Atomoxetine (N06BA09), and Lisdexamphetamine (N06BA12). ^b^ Tourette syndrome and chronic tic disorder were identified following the algorithm described in Rück et al.^35^ (2015). | | | |

**Supplementary Table 2.** Characteristics of the subcohort used in the survival analysis.

| **Characteristics** | **Obsessive-compulsive disorder,**  **n (%)** | **No record of obsessive-compulsive disorder,**  **n (%)** |
| --- | --- | --- |
| Total | 89 (0.6) | 15,163 (99.4) |
| Sex^a^ |  |  |
| Females | 58 (65.2) | 8,304 (54.8) |
| Males | 31 (34.8) | 6,859 (45.2) |
| Birth year^b^ |  |  |
| 1993-1994 | 18 (20.2) | 1,500 (9.9) |
| 1995-1997 | 28 (31.5) | 3,939 (26.0) |
| 1998-2000 | 23 (25.8) | 3,732 (24.6) |
| 2001-2003 | 16 (18.0) | 3,733 (24.6) |
| 2004-2006 | 4 (4.5) | 2,259 (14.9) |
| Age first OCD diagnosis |  |  |
| Mean (SD) | 18.7 (2.5) |  |
| Follow-up time^b^ |  |  |
| Mean (SD), years | 3.7 (2.5) | 6.3 (3.5) |
| Bullying victimization factor^c^ |  |  |
| Mean (SD) | 0.3 (1.3) | -0.0017 (0.9) |
| Bullying victimization^c,d^ |  |  |
| Yes | 7 (7.9) | 516 (3.4) |
| No | 82 (92.1) | 14,647 (96.6) |
| OCS at age 18 (BOCS) ^b,e^ |  |  |
| Mean (SD) | 5.8 (3.0) | 1.8 (2.2) |
| OCS at age 24 (OCI-R) ^b,f^ |  |  |
| Mean (SD) | 14.6 (8.6) | 7.8 (6.7) |
| ^a^ p-values not significant from either a Student t-test (continuous) or a Chi-square test (categorical) comparing OCD vs non-recorded OCD groups. ^b^ p-values significant at p<0.001. ^c^ p-values significant at p<0.05. ^d^ Cut-off for bullying victimization as in Solberg & Olweus^32^ (2003). ^e^ For the sub-cohort of individuals with OCS in CATSS-18. ^f^ For the sub-cohort of individuals with OCS in CATSS-24.  Abbreviations: BOCS, Brief Obsessive Compulsive Scale; OCD, obsessive-compulsive disorder; OCI-R, Obsessive-Compulsive Inventory-Revised; OCS, obsessive-compulsive symptoms; MZ, monozygotic; DZ, dizygotic; SD, standard deviation | | |

**Supplementary Table 3.** Survival analyses of the association between bullying victimization and obsessive-compulsive disorder diagnosed after age 15.

|  | **HR (95% CI)** | | |
| --- | --- | --- | --- |
|  | **Population level** | **Within twin comparisons** | |
|  | **Minimally adjusted^a^** | **Within DZ^b^** | **Within MZ** |
| HR per 1SD increase in the bullying victimization factor score | **1.21 (1.09–1.36)** | 1.50 (0.93–2.40) | 0.84 (0.48–1.47) |
|  |  | 4,408 pairs (47 OCD discordant) | 2,043 pairs (23 OCD discordant) |
| ^a^ Model adjusted for sex and year of birth.  ^b^ Model adjusted for sex.  Note: Bold figures indicate statistically significant (p<0.05).  Abbreviations: CI, confidence interval; HR, hazard ratio; OCD, obsessive compulsive disorder; MZ, monozygotic; DZ, dizygotic; SD, standard deviation. | | | |
